# Ethyl *ent*-15α-[(2-meth­oxy­benz­yloxy)meth­yl]-16-oxobeyeran-20-oate

**DOI:** 10.1107/S1600536812001833

**Published:** 2012-01-21

**Authors:** Ya Wu, Xia Wang, Jian-hong Gong, Chang-yong Wei, Jing-chao Tao

**Affiliations:** a Pharmacy College, Henan University of Traditional Chinese Medicine, Zhengzhou 450008, People’s Republic of China; b Department of Chemistry, New Drug Research & Development Center, Zhengzhou University, Zhengzhou 450052, People’s Republic of China

## Abstract

The title compound, C_31_H_44_O_5_, was synthesized from isostev­iol (systematic name: *ent*-16-ketobeyeran-19-oic acid). In the mol­ecule, the three six-membered rings adopt chair conformations and the stereochemistry of the *A*/*B* and *B*/*C* ring junctions are *trans*. The five-membered ring *D* adopts an envelope conformation with the methyl­ene C atom as the flap.

## Related literature

For background to isosteviol derivatives, see: Kinghorn *et al.* (1984 ▶); Yasukawa *et al.* (2002 ▶); Lin *et al.* (2004 ▶); Roy *et al.* (2007 ▶); Li *et al.* (2011 ▶). For a related structure, see: Shi (2010 ▶).
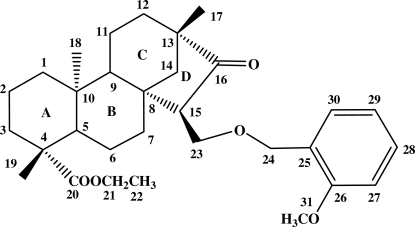


## Experimental

### 

#### Crystal data

C_31_H_44_O_5_
*M* *_r_* = 496.66
Orthorhombic, 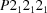

*a* = 8.7047 (17) Å
*b* = 10.749 (2) Å
*c* = 29.653 (6) Å
*V* = 2774.5 (9) Å^3^
*Z* = 4
Mo *K*α radiation
μ = 0.08 mm^−1^
*T* = 293 K
0.20 × 0.18 × 0.17 mm

#### Data collection

Rgaku R-AXIS-IV diffractometer
Absorption correction: multi-scan (*RAXIS*; Rigaku, 2004 ▶) *T*_min_ = 0.984, *T*_max_ = 0.987
8377 measured reflections
2876 independent reflections
2419 reflections with *I* > 2σ(*I*)
*R*_int_ = 0.102

#### Refinement

*R*[*F* ^2^ > 2σ(*F* ^2^)] = 0.065
*wR*(*F* ^2^) = 0.172
*S* = 1.08
2876 reflections
326 parameters
H-atom parameters constrained
Δρ_max_ = 0.21 e Å^−3^
Δρ_min_ = −0.19 e Å^−3^

### 

Data collection: *RAXIS* (Rigaku, 2004 ▶); cell refinement: *RAXIS*; data reduction: *RAXIS*; program(s) used to solve structure: *SHELXS97* (Sheldrick, 2008 ▶); program(s) used to refine structure: *SHELXL97* (Sheldrick, 2008 ▶); molecular graphics: *SHELXTL* (Sheldrick, 2008 ▶); software used to prepare material for publication: *SHELXL97*.

## Supplementary Material

Crystal structure: contains datablock(s) global, I. DOI: 10.1107/S1600536812001833/hb6600sup1.cif

Structure factors: contains datablock(s) I. DOI: 10.1107/S1600536812001833/hb6600Isup2.hkl

Additional supplementary materials:  crystallographic information; 3D view; checkCIF report

## Figures and Tables

**Fig. 1.**
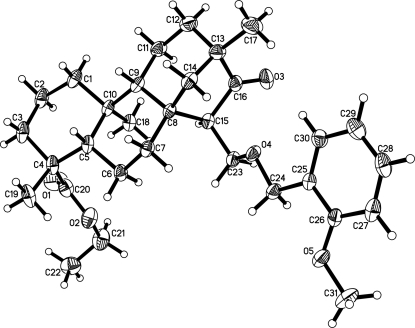
View of the title compound, showing 30% probability ellipsolids.

## supplementary crystallographic information

### Comment

Isosteviol (*ent*-16-ketobeyeran-19-oic acid 1) is a tetracyclic diterpenoid with a beyerane skeleton, obtained by acid hydrolysis of stevioside (Kinghorn *et al.*, 1984). In recent years, isosteviol derivatives have attracted scientific attention because of their remarkably broad spectrum of biological activities including anti-inflammatory, glucocorticoid agonist, antihypertension, antitumor, antiproliferation and inhibition of *ent*-kaurene synthase (Roy *et al.*, 2007; Li *et al.*, 2011; Yasukawa, *et al.*, 2002). Especially, Lin and co-workers reported that isosteviol amide dimers had favorable antibacterial effects and cytotoxicity (Lin, *et al.*, 2004), which prompted us to study new isosteviol derivatives to develop novel stronger antibacterial agents for therapeutic use. The title compound was synthesized from isosteviol. The molecule structure of the compound contains a fused four-ring system A/B/C/D and an aromatic ring (Fig. 1). The A/B ring and B/C junction are *trans*-fused, and C/D is *cis*-fused. The three six-membered rings adopt chair conformations, and the five-membered ring D adopts an envelope conformation with atom C14 displaced from the C8/C15/C16/C13 plane by 0.173 (5) Å. The C—C—C angles within the aromatic moiety cover a range 118.9 (4) - 121.4 (5) °.

### Experimental

The title compound was synthesized *via* esterification, Tollens reaction, 1,5-hydride shift from isosteviol, a kind of tetracyclo-diterpene, which has the skeleton of beyrane. To a stirred solution of ethyl-*ent*-15α-hydroxymethyl-16β-hydroxybeyeran-20-oate (0.378 g, 1 mmol) and ο-methoxybenzaldehyde (0.150 g, 1.1 mmol) in acetonitrile (10 mL) was added sulfuric acid (0.1 mmol). After stirring for 4 h at room temperature, the mixture was concentrated under vacuum and extracted with CHCl_3_ and H_2_O, at last the organic was washed with saturated NaCl aqueous solution, dried with MgSO_4_ and concentrated under vacuum. The residue was purified by column chromatography on silica (petroleum ether/ethyl acetate = 7:1, *v*/*v*) to give product (0.397 g, 80%). Colourless prisms were obtained by slow evaporation of an acetone solution.

### Refinement

Anomalous dispersion was negligible and Friedel pairs were merged before refinement. H atoms were generated geometrically and refined as riding atoms with C-H = 0.93Å and Uiso(H) = 1.2 times Ueq(C).

### Crystal data

**Table:**

C_31_H_44_O_5_	*D*_x_ = 1.189 Mg m^−^^3^
*M**_r_* = 496.66	Mo *K*α radiation, λ = 0.71073 Å
Orthorhombic, *P*2_1_2_1_2_1_	Cell parameters from 398 reflections
*a* = 8.7047 (17) Å	θ = 2.0–25.1°
*b* = 10.749 (2) Å	µ = 0.08 mm^−^^1^
*c* = 29.653 (6) Å	*T* = 293 K
*V* = 2774.5 (9) Å^3^	Prism, colorless
*Z* = 4	0.20 × 0.18 × 0.17 mm
*F*(000) = 1080	

### Data collection

**Table:**

Rgaku R-AXIS-IV diffractometer	2876 independent reflections
Radiation source: fine-focus sealed tube	2419 reflections with *I* > 2σ(*I*)
graphite	*R*_int_ = 0.102
Detector resolution: 0 pixels mm^-1^	θ_max_ = 25.5°, θ_min_ = 2.0°
Oscillation frames scans	*h* = −10→10
Absorption correction: multi-scan (RAXIS; Rigaku, 2004)	*k* = −13→0
*T*_min_ = 0.984, *T*_max_ = 0.987	*l* = −35→35
8377 measured reflections	

### Refinement

**Table:**

Refinement on *F*^2^	Secondary atom site location: difference Fourier map
Least-squares matrix: full	Hydrogen site location: inferred from neighbouring sites
*R*[*F*^2^ > 2σ(*F*^2^)] = 0.065	H-atom parameters constrained
*wR*(*F*^2^) = 0.172	*w* = 1/[σ^2^(*F*_o_^2^) + (0.0776*P*)^2^ + 0.8183*P*] where *P* = (*F*_o_^2^ + 2*F*_c_^2^)/3
*S* = 1.08	(Δ/σ)_max_ < 0.001
2876 reflections	Δρ_max_ = 0.21 e Å^−^^3^
326 parameters	Δρ_min_ = −0.19 e Å^−^^3^
0 restraints	Extinction correction: *SHELXL97* (Sheldrick, 2008), Fc^*^=kFc[1+0.001xFc^2^λ^3^/sin(2θ)]^-1/4^
Primary atom site location: structure-invariant direct methods	Extinction coefficient: 0.023 (3)

### Special details

**Table:**

Geometry. All e.s.d.'s (except the e.s.d. in the dihedral angle between two l.s. planes) are estimated using the full covariance matrix. The cell e.s.d.'s are taken into account individually in the estimation of e.s.d.'s in distances, angles and torsion angles; correlations between e.s.d.'s in cell parameters are only used when they are defined by crystal symmetry. An approximate (isotropic) treatment of cell e.s.d.'s is used for estimating e.s.d.'s involving l.s. planes.
Refinement. Refinement of *F*^2^ against ALL reflections. The weighted *R*-factor *wR* and goodness of fit *S* are based on *F*^2^, conventional *R*-factors *R* are based on *F*, with *F* set to zero for negative *F*^2^. The threshold expression of *F*^2^ > σ(*F*^2^) is used only for calculating *R*-factors(gt) *etc*. and is not relevant to the choice of reflections for refinement. *R*-factors based on *F*^2^ are statistically about twice as large as those based on *F*, and *R*- factors based on ALL data will be even larger.

### Fractional atomic coordinates and isotropic or equivalent isotropic displacement parameters (Å^2^)

**Table:**

	*x*	*y*	*z*	*U*_iso_*/*U*_eq_	
C1	0.6951 (6)	1.1089 (5)	−0.18318 (14)	0.0701 (13)	
H1A	0.6694	1.0227	−0.1892	0.084*	
H1B	0.5999	1.1531	−0.1775	0.084*	
C2	0.7708 (7)	1.1638 (5)	−0.22472 (15)	0.0803 (16)	
H2A	0.7039	1.1526	−0.2506	0.096*	
H2B	0.7862	1.2523	−0.2203	0.096*	
C3	0.9233 (7)	1.1021 (6)	−0.23378 (15)	0.0860 (17)	
H3A	0.9714	1.1433	−0.2592	0.103*	
H3B	0.9047	1.0164	−0.2424	0.103*	
C4	1.0369 (6)	1.1032 (4)	−0.19387 (14)	0.0654 (13)	
C5	0.9523 (6)	1.0518 (4)	−0.15150 (13)	0.0550 (11)	
H5A	0.9242	0.9668	−0.1601	0.066*	
C6	1.0479 (5)	1.0346 (4)	−0.10860 (13)	0.0571 (11)	
H6A	1.1468	0.9987	−0.1163	0.068*	
H6B	1.0657	1.1148	−0.0946	0.068*	
C7	0.9642 (5)	0.9495 (4)	−0.07574 (13)	0.0535 (10)	
H7A	0.9506	0.8687	−0.0898	0.064*	
H7B	1.0278	0.9380	−0.0492	0.064*	
C8	0.8081 (5)	0.9983 (3)	−0.06108 (12)	0.0455 (9)	
C9	0.7129 (5)	1.0364 (4)	−0.10343 (13)	0.0520 (10)	
H9A	0.6891	0.9574	−0.1183	0.062*	
C10	0.7951 (5)	1.1146 (4)	−0.14039 (12)	0.0524 (10)	
C11	0.5552 (6)	1.0883 (5)	−0.08938 (16)	0.0675 (12)	
H11A	0.5689	1.1719	−0.0778	0.081*	
H11B	0.4902	1.0935	−0.1159	0.081*	
C12	0.4735 (6)	1.0101 (6)	−0.05366 (19)	0.0787 (15)	
H12A	0.4295	0.9371	−0.0679	0.094*	
H12B	0.3900	1.0583	−0.0409	0.094*	
C13	0.5823 (5)	0.9684 (5)	−0.01536 (16)	0.0644 (12)	
C14	0.7142 (5)	0.8984 (4)	−0.03641 (15)	0.0609 (11)	
H14A	0.6770	0.8361	−0.0574	0.073*	
H14B	0.7758	0.8578	−0.0135	0.073*	
C15	0.8105 (5)	1.1029 (3)	−0.02504 (12)	0.0477 (9)	
H15A	0.8071	1.1837	−0.0403	0.057*	
C16	0.6624 (5)	1.0851 (4)	0.00128 (14)	0.0560 (11)	
C17	0.4945 (7)	0.9018 (7)	0.0217 (2)	0.102 (2)	
H17A	0.5646	0.8766	0.0450	0.153*	
H17B	0.4446	0.8297	0.0094	0.153*	
H17C	0.4188	0.9569	0.0342	0.153*	
C18	0.8167 (6)	1.2505 (4)	−0.12605 (14)	0.0586 (11)	
H18A	0.7184	1.2867	−0.1195	0.088*	
H18B	0.8648	1.2960	−0.1501	0.088*	
H18C	0.8804	1.2539	−0.0997	0.088*	
C19	1.1743 (7)	1.0177 (5)	−0.20565 (17)	0.0872 (17)	
H19A	1.2266	1.0499	−0.2317	0.131*	
H19B	1.1375	0.9353	−0.2120	0.131*	
H19C	1.2441	1.0148	−0.1806	0.131*	
C20	1.1039 (7)	1.2327 (5)	−0.18884 (16)	0.0714 (14)	
C21	1.2797 (9)	1.3612 (6)	−0.1511 (2)	0.104 (2)	
H21A	1.3269	1.3646	−0.1215	0.125*	
H21B	1.2007	1.4248	−0.1523	0.125*	
C22	1.3986 (7)	1.3888 (7)	−0.1861 (3)	0.107 (2)	
H22A	1.4410	1.4700	−0.1808	0.160*	
H22B	1.3523	1.3865	−0.2154	0.160*	
H22C	1.4789	1.3278	−0.1844	0.160*	
C23	0.9449 (5)	1.1021 (4)	0.00794 (13)	0.0568 (11)	
H23A	1.0414	1.1025	−0.0084	0.068*	
H23B	0.9413	1.1755	0.0269	0.068*	
C24	1.0648 (5)	0.9751 (5)	0.06169 (15)	0.0675 (13)	
H24A	1.0982	1.0544	0.0739	0.081*	
H24B	1.1475	0.9418	0.0434	0.081*	
C25	1.0298 (5)	0.8865 (4)	0.09980 (13)	0.0531 (10)	
C26	1.1467 (6)	0.8589 (4)	0.12974 (14)	0.0591 (11)	
C27	1.1178 (8)	0.7817 (5)	0.16676 (16)	0.0742 (15)	
H27A	1.1955	0.7641	0.1873	0.089*	
C28	0.9741 (8)	0.7323 (5)	0.17255 (18)	0.0797 (16)	
H28A	0.9548	0.6803	0.1970	0.096*	
C29	0.8599 (7)	0.7585 (5)	0.14303 (18)	0.0794 (15)	
H29A	0.7626	0.7245	0.1471	0.095*	
C30	0.8885 (6)	0.8363 (4)	0.10678 (15)	0.0628 (12)	
H30A	0.8094	0.8545	0.0868	0.075*	
C31	1.4097 (7)	0.8958 (7)	0.1493 (2)	0.107 (2)	
H31A	1.4990	0.9357	0.1370	0.160*	
H31B	1.4302	0.8088	0.1535	0.160*	
H31C	1.3848	0.9328	0.1779	0.160*	
O1	1.0710 (5)	1.3189 (4)	−0.21179 (17)	0.1141 (16)	
O2	1.2094 (5)	1.2413 (3)	−0.15713 (12)	0.0875 (11)	
O3	0.6178 (5)	1.1533 (4)	0.03115 (11)	0.0841 (11)	
O4	0.9339 (3)	0.9930 (3)	0.03482 (9)	0.0578 (8)	
O5	1.2847 (4)	0.9107 (4)	0.11943 (11)	0.0820 (11)	

### Atomic displacement parameters (Å^2^)

**Table:**

	*U*^11^	*U*^22^	*U*^33^	*U*^12^	*U*^13^	*U*^23^
C1	0.095 (3)	0.067 (3)	0.048 (2)	0.006 (3)	−0.029 (2)	0.004 (2)
C2	0.110 (4)	0.087 (3)	0.045 (2)	0.020 (3)	−0.023 (3)	0.003 (2)
C3	0.130 (5)	0.091 (4)	0.037 (2)	0.012 (4)	−0.007 (3)	−0.002 (2)
C4	0.097 (3)	0.060 (3)	0.040 (2)	0.017 (3)	0.003 (2)	0.001 (2)
C5	0.081 (3)	0.046 (2)	0.038 (2)	0.009 (2)	0.000 (2)	−0.0034 (17)
C6	0.068 (3)	0.061 (2)	0.042 (2)	0.014 (2)	0.001 (2)	0.005 (2)
C7	0.067 (3)	0.048 (2)	0.045 (2)	0.010 (2)	−0.011 (2)	0.0031 (18)
C8	0.061 (2)	0.0369 (18)	0.0387 (19)	−0.0005 (19)	−0.0095 (18)	0.0003 (15)
C9	0.064 (2)	0.045 (2)	0.047 (2)	0.001 (2)	−0.015 (2)	−0.0057 (17)
C10	0.077 (3)	0.044 (2)	0.0363 (19)	0.004 (2)	−0.014 (2)	−0.0018 (16)
C11	0.063 (3)	0.077 (3)	0.062 (3)	0.002 (3)	−0.018 (2)	0.009 (2)
C12	0.063 (3)	0.096 (4)	0.077 (3)	−0.006 (3)	−0.012 (3)	0.008 (3)
C13	0.057 (3)	0.071 (3)	0.065 (3)	−0.008 (2)	−0.008 (2)	0.014 (2)
C14	0.079 (3)	0.048 (2)	0.056 (2)	−0.011 (2)	−0.014 (2)	0.006 (2)
C15	0.066 (2)	0.0367 (18)	0.0406 (19)	−0.001 (2)	−0.0022 (19)	0.0032 (16)
C16	0.060 (2)	0.063 (3)	0.045 (2)	0.012 (2)	−0.0022 (19)	0.009 (2)
C17	0.076 (3)	0.129 (5)	0.100 (4)	−0.024 (4)	0.000 (3)	0.040 (4)
C18	0.081 (3)	0.046 (2)	0.049 (2)	0.009 (2)	−0.001 (2)	0.0032 (18)
C19	0.122 (5)	0.081 (3)	0.059 (3)	0.032 (3)	0.030 (3)	−0.003 (3)
C20	0.089 (4)	0.068 (3)	0.058 (3)	0.014 (3)	0.007 (3)	0.016 (2)
C21	0.146 (6)	0.072 (3)	0.093 (4)	−0.019 (4)	−0.001 (4)	−0.007 (3)
C22	0.094 (4)	0.090 (4)	0.137 (6)	0.006 (4)	−0.010 (4)	−0.002 (4)
C23	0.071 (3)	0.054 (2)	0.045 (2)	−0.021 (2)	−0.002 (2)	0.0006 (19)
C24	0.063 (3)	0.090 (3)	0.050 (2)	−0.013 (3)	−0.012 (2)	0.015 (2)
C25	0.064 (3)	0.055 (2)	0.040 (2)	0.002 (2)	0.000 (2)	0.0003 (18)
C26	0.071 (3)	0.065 (3)	0.041 (2)	0.008 (2)	−0.003 (2)	−0.006 (2)
C27	0.112 (4)	0.064 (3)	0.047 (2)	0.023 (3)	−0.010 (3)	−0.001 (2)
C28	0.110 (4)	0.071 (3)	0.058 (3)	0.013 (3)	0.019 (3)	0.016 (3)
C29	0.093 (4)	0.068 (3)	0.078 (3)	0.001 (3)	0.024 (3)	0.013 (3)
C30	0.071 (3)	0.062 (2)	0.055 (2)	−0.002 (2)	0.004 (2)	0.007 (2)
C31	0.097 (4)	0.124 (5)	0.099 (4)	0.009 (4)	−0.051 (4)	−0.005 (4)
O1	0.114 (3)	0.094 (3)	0.134 (4)	−0.007 (3)	−0.024 (3)	0.064 (3)
O2	0.128 (3)	0.067 (2)	0.067 (2)	0.001 (2)	−0.009 (2)	0.0072 (17)
O3	0.099 (3)	0.095 (2)	0.0579 (19)	0.024 (2)	0.0141 (19)	−0.0073 (19)
O4	0.0654 (17)	0.0623 (17)	0.0456 (15)	−0.0147 (15)	−0.0147 (13)	0.0140 (13)
O5	0.072 (2)	0.115 (3)	0.0582 (19)	−0.008 (2)	−0.0214 (17)	0.011 (2)

### Geometric parameters (Å, °)

**Table:**

C1—C2	1.516 (7)	C15—H15A	0.9800
C1—C10	1.540 (5)	C16—O3	1.213 (5)
C1—H1A	0.9700	C17—H17A	0.9600
C1—H1B	0.9700	C17—H17B	0.9600
C2—C3	1.508 (7)	C17—H17C	0.9600
C2—H2A	0.9700	C18—H18A	0.9600
C2—H2B	0.9700	C18—H18B	0.9600
C3—C4	1.542 (7)	C18—H18C	0.9600
C3—H3A	0.9700	C19—H19A	0.9600
C3—H3B	0.9700	C19—H19B	0.9600
C4—C20	1.517 (8)	C19—H19C	0.9600
C4—C19	1.549 (7)	C20—O1	1.184 (6)
C4—C5	1.558 (6)	C20—O2	1.317 (6)
C5—C6	1.531 (6)	C21—O2	1.438 (7)
C5—C10	1.560 (6)	C21—C22	1.495 (9)
C5—H5A	0.9800	C21—H21A	0.9700
C6—C7	1.522 (6)	C21—H21B	0.9700
C6—H6A	0.9700	C22—H22A	0.9600
C6—H6B	0.9700	C22—H22B	0.9600
C7—C8	1.520 (6)	C22—H22C	0.9600
C7—H7A	0.9700	C23—O4	1.422 (5)
C7—H7B	0.9700	C23—H23A	0.9700
C8—C14	1.534 (6)	C23—H23B	0.9700
C8—C15	1.552 (5)	C24—O4	1.403 (5)
C8—C9	1.560 (5)	C24—C25	1.509 (6)
C9—C11	1.538 (7)	C24—H24A	0.9700
C9—C10	1.556 (6)	C24—H24B	0.9700
C9—H9A	0.9800	C25—C30	1.359 (6)
C10—C18	1.533 (6)	C25—C26	1.382 (6)
C11—C12	1.527 (7)	C26—O5	1.359 (6)
C11—H11A	0.9700	C26—C27	1.398 (7)
C11—H11B	0.9700	C27—C28	1.369 (8)
C12—C13	1.545 (7)	C27—H27A	0.9300
C12—H12A	0.9700	C28—C29	1.354 (8)
C12—H12B	0.9700	C28—H28A	0.9300
C13—C14	1.508 (7)	C29—C30	1.384 (7)
C13—C16	1.518 (7)	C29—H29A	0.9300
C13—C17	1.518 (7)	C30—H30A	0.9300
C14—H14A	0.9700	C31—O5	1.413 (6)
C14—H14B	0.9700	C31—H31A	0.9600
C15—C16	1.519 (6)	C31—H31B	0.9600
C15—C23	1.525 (6)	C31—H31C	0.9600
			
C2—C1—C10	114.1 (4)	H14A—C14—H14B	108.9
C2—C1—H1A	108.7	C16—C15—C23	108.7 (3)
C10—C1—H1A	108.7	C16—C15—C8	104.5 (3)
C2—C1—H1B	108.7	C23—C15—C8	116.6 (3)
C10—C1—H1B	108.7	C16—C15—H15A	108.9
H1A—C1—H1B	107.6	C23—C15—H15A	108.9
C3—C2—C1	110.9 (4)	C8—C15—H15A	108.9
C3—C2—H2A	109.5	O3—C16—C13	126.1 (4)
C1—C2—H2A	109.5	O3—C16—C15	124.8 (4)
C3—C2—H2B	109.5	C13—C16—C15	109.1 (4)
C1—C2—H2B	109.5	C13—C17—H17A	109.5
H2A—C2—H2B	108.1	C13—C17—H17B	109.5
C2—C3—C4	115.1 (4)	H17A—C17—H17B	109.5
C2—C3—H3A	108.5	C13—C17—H17C	109.5
C4—C3—H3A	108.5	H17A—C17—H17C	109.5
C2—C3—H3B	108.5	H17B—C17—H17C	109.5
C4—C3—H3B	108.5	C10—C18—H18A	109.5
H3A—C3—H3B	107.5	C10—C18—H18B	109.5
C20—C4—C3	109.2 (4)	H18A—C18—H18B	109.5
C20—C4—C19	105.7 (5)	C10—C18—H18C	109.5
C3—C4—C19	108.5 (4)	H18A—C18—H18C	109.5
C20—C4—C5	115.3 (4)	H18B—C18—H18C	109.5
C3—C4—C5	108.2 (4)	C4—C19—H19A	109.5
C19—C4—C5	109.7 (4)	C4—C19—H19B	109.5
C6—C5—C4	117.1 (4)	H19A—C19—H19B	109.5
C6—C5—C10	110.7 (3)	C4—C19—H19C	109.5
C4—C5—C10	115.6 (3)	H19A—C19—H19C	109.5
C6—C5—H5A	103.8	H19B—C19—H19C	109.5
C4—C5—H5A	103.8	O1—C20—O2	121.6 (5)
C10—C5—H5A	103.8	O1—C20—C4	124.6 (5)
C7—C6—C5	110.2 (4)	O2—C20—C4	113.7 (4)
C7—C6—H6A	109.6	O2—C21—C22	112.8 (6)
C5—C6—H6A	109.6	O2—C21—H21A	109.0
C7—C6—H6B	109.6	C22—C21—H21A	109.0
C5—C6—H6B	109.6	O2—C21—H21B	109.0
H6A—C6—H6B	108.1	C22—C21—H21B	109.0
C8—C7—C6	113.8 (3)	H21A—C21—H21B	107.8
C8—C7—H7A	108.8	C21—C22—H22A	109.5
C6—C7—H7A	108.8	C21—C22—H22B	109.5
C8—C7—H7B	108.8	H22A—C22—H22B	109.5
C6—C7—H7B	108.8	C21—C22—H22C	109.5
H7A—C7—H7B	107.7	H22A—C22—H22C	109.5
C7—C8—C14	111.8 (3)	H22B—C22—H22C	109.5
C7—C8—C15	115.8 (3)	O4—C23—C15	108.2 (3)
C14—C8—C15	100.7 (3)	O4—C23—H23A	110.1
C7—C8—C9	109.6 (3)	C15—C23—H23A	110.1
C14—C8—C9	106.6 (3)	O4—C23—H23B	110.1
C15—C8—C9	111.8 (3)	C15—C23—H23B	110.1
C11—C9—C10	113.9 (3)	H23A—C23—H23B	108.4
C11—C9—C8	110.6 (3)	O4—C24—C25	110.3 (4)
C10—C9—C8	117.7 (3)	O4—C24—H24A	109.6
C11—C9—H9A	104.3	C25—C24—H24A	109.6
C10—C9—H9A	104.3	O4—C24—H24B	109.6
C8—C9—H9A	104.3	C25—C24—H24B	109.6
C18—C10—C1	109.6 (3)	H24A—C24—H24B	108.1
C18—C10—C9	112.1 (3)	C30—C25—C26	118.9 (4)
C1—C10—C9	107.4 (3)	C30—C25—C24	123.2 (4)
C18—C10—C5	111.3 (4)	C26—C25—C24	117.9 (4)
C1—C10—C5	107.7 (3)	O5—C26—C25	114.7 (4)
C9—C10—C5	108.6 (3)	O5—C26—C27	125.3 (5)
C12—C11—C9	113.8 (4)	C25—C26—C27	119.9 (5)
C12—C11—H11A	108.8	C28—C27—C26	119.5 (5)
C9—C11—H11A	108.8	C28—C27—H27A	120.2
C12—C11—H11B	108.8	C26—C27—H27A	120.2
C9—C11—H11B	108.8	C29—C28—C27	120.6 (5)
H11A—C11—H11B	107.7	C29—C28—H28A	119.7
C11—C12—C13	112.6 (4)	C27—C28—H28A	119.7
C11—C12—H12A	109.1	C28—C29—C30	119.7 (5)
C13—C12—H12A	109.1	C28—C29—H29A	120.2
C11—C12—H12B	109.1	C30—C29—H29A	120.2
C13—C12—H12B	109.1	C25—C30—C29	121.4 (5)
H12A—C12—H12B	107.8	C25—C30—H30A	119.3
C14—C13—C16	101.3 (3)	C29—C30—H30A	119.3
C14—C13—C17	116.6 (4)	O5—C31—H31A	109.5
C16—C13—C17	112.7 (4)	O5—C31—H31B	109.5
C14—C13—C12	107.9 (4)	H31A—C31—H31B	109.5
C16—C13—C12	106.3 (4)	O5—C31—H31C	109.5
C17—C13—C12	111.2 (4)	H31A—C31—H31C	109.5
C13—C14—C8	104.7 (3)	H31B—C31—H31C	109.5
C13—C14—H14A	110.8	C20—O2—C21	116.6 (4)
C8—C14—H14A	110.8	C24—O4—C23	112.1 (3)
C13—C14—H14B	110.8	C26—O5—C31	119.5 (4)
C8—C14—H14B	110.8		
